# Radiotherapy Quality Assurance for Head and Neck Squamous Cell Carcinoma

**DOI:** 10.3389/fonc.2020.00282

**Published:** 2020-03-12

**Authors:** Dirk Van Gestel, Tatiana Dragan, Vincent Grégoire, Mererid Evans, Volker Budach

**Affiliations:** ^1^Department of Radiation Oncology Head and Neck Unit, Institut Jules Bordet, Université Libre de Bruxelles, Brussels, Belgium; ^2^Radiation Oncology Departement, Léon Bérard Cancer Center, Lyon, France; ^3^Department of Clinical Oncology, Velindre University NHS Trust, Cardiff, United Kingdom; ^4^Departments of Radiation Oncology, Charité University Medicine Berlin, Berlin, Germany

**Keywords:** best practice, quality assurance, radiotherapy, IMRT, head and neck cancer, squamous cell carcinoma

## Abstract

The impact of radiotherapy (RT) quality assurance (QA) has been demonstrated by numerous studies and is particularly important for head and neck cancer (HNC) treatment due to the complexity of RT target volumes in this region and the multiple adjacent organs at risk. The RT planning process includes many critical steps including interpretation of diagnostic imaging, image fusion, target volume delineation (tumor, lymph nodes, and organs at risk), and planning. Each step has become highly complex, and precise and rigorous QA throughout the planning process is essential. The ultimate aim is to precisely deliver radiation dose to the target, maximizing the tumor dose and minimizing the dose to surrounding organs at risk, in order to improve the therapeutic index. It is imperative that RT QA programs should systematically control all aspects of the RT planning pathway and include regular end-to-end tests and external audits. However, comprehensive QA should not be limited to RT and should, where possible, also be implemented for surgery, systemic therapy, pathology, as well as other aspects involved in the interdisciplinary treatment of HNC.

## Introduction

Photon-based radiotherapy (RT) techniques have evolved enormously since the introduction of computerized axial tomography (CT) scanning in RT planning 30 years ago. Since then, external beam RT has evolved from two-dimensional (2D) conventional RT to 3D conformal RT, then static beam intensity-modulated RT (IMRT), and ultimately to rotational IMRT or volumetric modulated arc therapy (VMAT) (1).

IMRT is a technique that combines irradiation beams with non-uniform fluence intensity to generate steep dose gradients even in target volumes (TVs) with a concave shape (2). As a direct consequence, TVs are treated more homogeneously and with a better sparing of the nearby organs at risk (OARs), in comparison with the classical 2D or 3D RT techniques. This better sparing of the OARs is particularly relevant in areas of the body where there are relatively radioresistant TVs in close vicinity to radiosensitive OARs, such as in the head and neck area. Consequently, IMRT has become standard of care for the treatment of head and neck cancer (HNC) based on a proven superiority over 3D conformal RT in terms of prevention of xerostomia (3–7). In the last decade, another emerging technique, intensity-modulated proton therapy (IMPT), has been tested for its potential to reduce side effects in HNC patients, beyond what IMRT using photons can achieve (8). IMPT can be more sensitive to changes in patient setup, CT scan values, and patient anatomy than IMRT because of uncertainties surrounding the precise location of the distal edge of the Bragg peak. The parallel development of high-level 3D image guidance to allow accurate on-treatment verification, including cone beam CT scan (CBCT), megavoltage CT (MV-CT), or kilovoltage CT (kV-CT), and MRI Linacs has been indispensable to allow new RT treatment techniques to reach their maximal potential (1).

The technological revolution described above has significantly increased the complexity of RT, leading to increased efforts to ensure the quality of RT planning and delivery (9). Proactive RT quality assurance (QA) programs and extended guidelines have been developed for clinical trials as well as routine practice, which should nowadays be fully implemented in every RT department. The realization that QA can have a major impact on the outcome, especially in HNC, highlights the importance of such endeavors.

In this article, we will give an overview of the recent history of RT QA, its impact on outcome in HNC patients, and the measures that can be taken to optimize RT in the management of HNC.

## Early Data on the Impact of RT QA

The process of RT planning and treatment is complex and includes many steps including consultation with the patient, interpretation of diagnostic imaging, TV delineation, treatment planning, treatment delivery, and patient follow up. Each of these steps must be seamlessly integrated into the RT pathway and needs careful QA.

In 2001, the Radiological Physics Center at the MD Anderson Cancer Center (USA) compared, planned, and delivered dose in a phantom study of IMRT in HNC and found a 43% failure rate in obtaining the 5%/3 mm criteria (i.e., the measured dose at a certain point being within 5% or 3 mm of the planned dose) (10). Depending on the shape/steepness of the dose–response curves, this could potentially translate, in a patient, into significant differences in tumor control and/or toxicity. More recently, BELdART (BELgian dosimetry Audits in Radio Therapy) found one Belgian center to have a passing rate of <90% in their gamma 3%/3 mm measurements, highlighting the need for regular external audits (11).

In 2003, Khalil et al. published data on compliance to the prescribed dose-fractionation schedule and overall treatment time in five randomized controlled trials of altered fractionation RT for HNC (12). Only 30% of patients appeared to have been treated within the calculated ideal overall treatment time, a well-known factor in the local control of HNC (13, 14). Centers varied significantly in their compliance and the authors concluded that poor compliance could affect the outcome of these trials.

## Impact of RT QA on Outcome in HNC

The severe, often deadly RT accidents listed by Knöös et al. have received significant publicity in the past, but have become extremely rare today because of QA (15). Moreover, several studies have shown that the quality of RT can have a positive impact on outcome in patients with HNC.

Fairchild and colleagues reviewed 17 multicenter studies (1980–2012) including five studies dealing with HNC: four Radiation Therapy Oncology Group (RTOG) and one Trans Tasman Radiation Oncology Group (TROG) study (16). In four HNC trials, patients had inferior outcomes when RT was judged to be inadequate compared to when it was adequate. Three HNC trials suggested that RT that was deemed to be compliant with the study protocol significantly increased overall survival. The impact of QA on outcome in HNC from selected studies is presented in Table 1.

**Table 1:** The impact of QA on outcome in HNC from selected studies.

**References**	**Organization**	***N***	**Main outcome and QA issue**
Pajak et al. (17)	RTOG 7913 RTOG 7915	210 306	3-year OS 13% if unacceptable deviation vs. 26% if acceptable (*p* = 0.01)
Eisbruch et al. (18)	RTOG 0022	69	2/4 cases with major deviations (PTV dose) had LRR vs. 3/49 if no major deviation in PTV dose (*p* = 0.04)
Peters et al. (19)	TROG 0202	861	2-year OS 50% if major deviation (*n* = 87) vs. 70% if protocol compliant (*n* = 502) (*p* < 0.001)
Wuthrick et al. (20)	RTOG 0121	471	5-year OS 51% in low-accruing centers vs. 69.1% in high-accruing centers (*p* = 0.002)
Naghavi et al. (21)		1,390	3-year OS 57% in low-accruing centers vs. 72% in high-accruing centers (*p* < 0.001)

*OS, overall survival; PTV, planning target volume; LRR, locoregional relapse; RTOG, Radiation Therapy Oncology Group; TROG, Trans Tasman Radiation Oncology Group*.

The landmark study that demonstrated the impact of QA on outcomes in HNC was the TROG 0202 study, a large international phase III trial, published by Peters and colleagues. They found that QA had a major impact on the outcome of HNC patients treated with chemo-RT (in the pre IMRT era) (19). In the study, 12% of patients with RT plans in which there were major protocol violations (3% due to poor contouring and 5% due to poor plan preparation) had a 24% lower freedom from loco-regional failure rate (54% vs. 78%; *p* < 0.001) and a 20% reduction in overall survival (50% vs. 70%; *p* < 0.001) at 2 years follow-up, compared to those with RT plans that were fully compliant from the start. The authors concluded, “It is sobering to note that the value of good RT is substantially greater than the incremental gains that have been achieved with new drugs and/or biological.” Interestingly, the rate of major protocol violations per treatment center was inversely correlated with the number of patients enrolled by the center (<5 patients: 29.8%; >20 patients: 5.4%; *p* < 0.001). These data illustrate the importance of careful QA coupled with external audits for highly sophisticated RT techniques in HNC and highlight the need for centralized and experienced high patient throughput RT centers (22). Furthermore, when the investigators excluded data from the 12% of patients with major RT protocol violations from the trial analysis they found, contrary to the initial negative results for the whole group, there was a strong tendency for improved locoregional control in favor of the experimental tirapazamine arm (79% vs. 75% at 2 years; *p* = 0.067). This indicates the enormous potential impact of RT QA on the results of multicenter trials. Previous RT trials, which were negative, might have been positive and vice versa if RT QA was insufficient. This sobering message provides a tremendous incentive for improving standardized QA measures in our future clinical trials.

The above studies were conducted in in a non-IMRT population; however, IMRT has become the standard of care for the treatment of HNC since the publication of the PARSPORT study (5). Because of its increased complexity and sophistication, an even bigger impact of RT QA can be expected with IMRT.

Boero et al. retrospectively analyzed 6,212 HNC patients on the Surveillance, Epidemiology, and End Results (SEER) population-based cancer registry and found that in the case of IMRT, the risk of all-cause mortality decreased by 21% for every additional five patients treated per provider per year, because of a decrease in HNC-specific mortality and the risk of aspiration pneumonia. No such relationship was found for conventional RT (23). Important additional evidence that patients with advanced HNC should be treated in high-volume HNC centers for optimal survival outcomes is provided by two recently published retrospective analyses using the National Cancer Database from United States. The first study included 46,567 patients diagnosed with locally advanced invasive squamous cell carcinomas of the oropharynx, larynx, and hypopharynx and undergoing definitive RT. The 5-year overall survival rate was 61.6% vs. 55.5%, respectively, for patients treated at high-volume facilities vs. lower-volume facilities (*p* < 0.001) (24). The second study, which focused on 4,469 patients with nasopharyngeal cancer, demonstrated that treatment at high-volume centers is an independent predictor of higher overall survival (HR, 0.85; 95% CI, 0.75–0.96) (25).

## Importance of RT QA in Clinical Trials

Learning from the negative experience of the TROG 0202 study, the EORTC organized an extended “dummy run” for their phase III EORTC 22071-26071 study designed to evaluate the addition of panitumumab to adjuvant chemo-IMRT in locally advanced, resected squamous cell HNC (19, 26) A computed tomography dataset comprising one case of NHC was sent to the participating institutions and then compared with reference contours and protocol guidelines by six central reviewers. Of the 23 datasets, 13% of the GTV (gross tumor volume = macroscopic disease), 44% of the CTV (clinical TV = zone of possible microscopic extension), and 57% of the PTV (planning TV = margin for movement and setup uncertainty) contours were evaluated unacceptable (objectives and constraints defined per protocol and taking into account all available information along with ICRU recommendations) by the expert panel. Overall, only 13% of the sites that combined TVs were considered acceptable, 43.5% had minor deviations, and 43.5% were judged to have major deviations. Of all the sites, 74, 87, and 91% met the dose constraints for the low-dose, intermediate-dose, and high-dose volumes, respectively. Almost all deviations were found in the minimal dose constraints (D98 and D95%), i.e., an underdose of a part of the TV. No statistical correlation was found between the achievement of the dose constraints and the PTV contour evaluation by the experts. For the OARs, sites met the dose constraints for an average of three OARs out of six (often at the price of PTV coverage), and for most OARs (but not for the parotid glands), a significant correlation between the quality of the contouring and the sites' ability to respect the OAR's specific dose constraints (and thus their ability to limit the toxicity) was reported. They concluded that wide variations exist despite strict guidelines, confirming the complexities involved in developing and delivering QA for IMRT-based multicenter studies for HNC. Another phase III EORTC 1219–DAHANCA 29 intergroup trial designed to evaluate the influence of nimorazole in patients with locally advanced HNC when treated with accelerated RT in combination with chemotherapy provided a RT QA program for the participating centers (27). A pre-trial benchmark case was delineated and planned and prospectively centrally reviewed. Fifty-four submissions from 19 centers were reviewed. Nine (47%) centers needed to perform the delineation step twice and three (16%) centers repeated it three times before receiving approval. The authors highlighted the importance of clearly defined protocol guidelines to avoid unacceptable errors.

While strict adherence to ICRU 83 guidelines on “Prescribing, Recording, and Reporting Intensity-Modulated Photon-Beam Therapy” can address most of the (QA) issues required to obtain adequate dose distribution during planning and delivery, work is still required to achieve consensus and QA of contouring (28). In addition to the study by Fairchild et al. mentioned above, the PARSPORT study also found large differences in contouring in 3 out of 10 submissions due to lack of adherence to the trial guidelines (26, 29). The Swiss national “dummy run” study found that more precise radiological imaging could increase homogeneity in delineation of the GTV (30). Regarding the CTVs, international consensus guidelines have been developed for the delineation of the nodal and primary CTVs that are beneficial for harmonization in routine clinical practice and essential for clinical trial RT QA (31–36). However, in 2010, Rasch et al. reported considerable heterogeneity in CTV delineation among Dutch radiation oncologists, despite the publication of guidelines on CTV delineation by Gregoire et al. (31). Furthermore, in 2017, van der Veen et al. found large discrepancies in the selection of prophylactic nodal levels and CTVs delineated among Belgian centers (14/22) (37, 38), illustrating that continued efforts are required in training and education to improve standardization.

In addition to heterogeneity in TV delineation, Nelms et al. reported major variations in the sizes and shapes of OARs contoured by different radiation oncologists from international participating centers in an oropharyngeal cancer patient (39). In the meantime, Brouwers et al. published consensus guidelines on the contouring of HNC OARs, with the aim of reducing the heterogeneity of OAR contouring in clinical studies as well as in daily practice (40). Interestingly, this consensus was published after a delineation study of OARs by a panel of seven HNC RT experts that demonstrated significant differences in OAR contouring (coefficient of variance ranging from 12% for the parotid gland up to 56% for the glottis larynx) (41).

As a result of the heterogeneity outlined above, the EORTC HNC group and other groups including the UK RT Trials QA (RTTQA) Group have further fine-tuned the quality control of their HNC trials by adding individual patient plan reviews to the pre-trial benchmark case. Each participating center is requested to send the planning CT of each of their enrolled patients to the QA RT platform for review of the TV selection and delineation. When approved, centers are then asked to send the planned dose distribution to the QA platform. Ideally, this should be done for every single patient. For pragmatic and cost reasons, it is often prospectively performed only for the first 5 or 10 patients. The plans will, however, be collected for all patients enrolled in the trial, allowing for retrospective evaluation of all cases.

## The Cost of QA

Data on the costs associated with RT QA are scarce due to the practical difficulties associated with carrying out economic studies in this field, in terms of cost calculation and efficacy data (42).

While one might expect more/higher-level QA to result in a higher global cost, the opposite may be true. In a simulated study, Weber et al. showed that increasing QA level in a prospective HNC trial translated into better overall survival and a decreased tumor recurrence rate (43). They found a positive association between the complexity of QA procedures and the patient's outcome, resulting in a lower general cost for more complex and thus more expensive QA, due to fewer recurrences and thus fewer costs for re-treatment. It is also possible to improve patient's outcomes parallel to the care process without incurring any additional costs. Simons et al. reported the cost-effectiveness and improvement in patient outcomes seen after reducing the waiting times to start treatment (crucial for HNC patients). In their new workflow, the reduction in waiting time varied from 5 days for patients treated for oropharyngeal or hypopharyngeal cancer to 22 days for laryngeal cancer patients resulting in 0.13 to 0.66 additional quality-adjusted life years (44).

The fact that higher QA costs often have to be paid for by the RT department/hospital while the benefits (improved outcomes) are seen by society/government might deter some RT centers from stepping up to implement a higher level of QA. Therefore, efforts should be made to better reimburse these treatment-specific higher QA costs.

## QA in the Routine Clinical Setting

Overall, the abovementioned studies confirm the complexity of IMRT-based multicenter studies and they stress the importance of adhering to strict QA procedures, not only in the framework of clinical trials but also in routine daily practice. When QA problems occur in studies involving motivated, well-informed RT departments guided by a detailed protocol, it is reasonable to assume that similar issues can occur in any RT department in the routine clinical setting that may or may not be identified. Therefore, consensus meetings and external audits with end-to-end testing of the whole RT process, in general, and of the QA, in particular, are of utmost importance (9, 15). Understanding the incidence, types, and reasons for variation in compliance in clinical trials contributes to the understanding of the application and limitation of RT QA in the routine clinical setting, and the training and lessons learnt from clinical trials tend to increase quality within daily practice. However, despite the move to include central individual patient contour (and dosimetry) review in recent EORTC studies, we do not yet have a technological solution to QA the most important variable in routine RT practice, i.e., TV delineation. Continuous education, practical sessions, peer review programs, automatization, and multidisciplinary contouring (e.g., with the radiologist and/or head and neck surgeon) are more important than ever to avoid geographical miss (9, 22, 45). Recent studies stress the importance of peer review. Bergamini et al. retrospectively analyzed 781 HNC patients of whom ~70% were referred for a second opinion. Following multidisciplinary evaluation, new staging examinations were requested in 49% of patients and treatment was modified in 10% (46). A recent review by McDowell and Corry stated that even in high-volume academic HNC institutions, major plan changes are not infrequent following peer review; errare humanum est (47). Therefore, peer review should be standard practice in all centers and there is a strong argument that centers without an adequate RT QA process should not offer treatments to patients with HNC.

Routine clinical QA should go further than verification of contouring, to include QA of the dose distribution and the delivery of the correct dose of radiation within the planned time frame, as routinely studied in the context of clinical trials (15). Routine QA should also include continuous training at all steps in the RT process, rigorous image fusion, precise patient setup, verification of treatment delivery using offline or ideally online image guidance (IGRT, image-guided RT), and careful follow-up looking for late side effects, recurrences, and second primaries. In terms of IGRT, Den et al. conducted a prospective study of 28 HNC patients (1,013 kV CBCT scans) highlighting the importance of daily imaging for treatment accuracy and margin size. They found that by using daily imaging, most of the PTV margins could be reduced by as much as 50% compared to the margins applied when using non-daily imaging (mediolaterally 1.6 vs. 3.9 mm; superioinferiorly 2.5 vs. 4.1 mm; anteroposteriorly 1.9 vs. 4.9 mm, respectively). This radius reduction corresponds to a much larger reduction in the volume of healthy tissue being irradiated (*V* = 4/3π*r*^3^) (48). Moreover, PTV margins should be based on the individual department's calculation of their setup margin of error; yet, in practice, many centers use PTV margins derived from the literature and implement non-daily image guidance protocols.

Maybe (one of) the abovementioned steps can explain the unexplained survival drop after 3 years in the TROG 0202 population who was made compliant or who had only minor protocol deviations compared to the patients fully treated by protocol from the start (19). In other words, the whole process from A to Z has to be optimal to get the best results for our HNC patients. The recent technological evolution in RT paralleled with the increasing awareness of the importance of QA, as described above, means that major efforts have and are still being made to improve QA at each step of the treatment pathway, not only for trials but also in daily practice (15).

## Importance of QA in Other Aspects of Treatment

Increasing awareness of the importance of QA and of centralization remains largely restricted to the RT aspect of HNC treatment. More and more data are converging to illustrate that the outcome of patients with HNC is better when performed in large volume centers compared to low volume centers (20, 21, 24, 25). The reason for this finding is likely multi-factorial, including not only the quality of RT planning and delivery, but also the quality and accuracy of other steps involved in tumor staging (e.g., pathology, imaging) and treatment (e.g., surgery, systemic treatment). Furthermore, proper integration of these steps into the patient care pathway is extremely important, as is the physician and hospital's capacity to react to changes and incidents occurring during the patient's journey through treatment.

## Conclusions

The increasing complexity and precision of modern RT techniques, particularly for HNC, means that rigorous QA is essential in every step of the RT pathway, in order to deliver the right dose in exactly the right place to optimize tumor control and minimize toxicity. Therefore, RT QA, in routine practice as well as in clinical trials, should include a clear program to systematically control each step in the pathway as well as regular end-to-end tests and external audits. Ideally, this QA should not be limited to RT, but should also encompass every aspect of the patient pathway, in order to fully realize the benefits associated with the delivery of safe, standardized, and high-quality patient care.

## Author Contributions

DV and TD wrote the first draft of the manuscript and VG and VB revised it critically for important intellectual content. ME edited the final manuscript. All authors approved the submitted version.

### Conflict of Interest

The authors declare that the research was conducted in the absence of any commercial or financial relationships that could be construed as a potential conflict of interest.
